# MTHFR *C677T* gene polymorphism and the severity of coronary lesions in acute coronary syndrome

**DOI:** 10.1097/MD.0000000000009044

**Published:** 2017-12-08

**Authors:** Miao-Nan Li, Hong-Ju Wang, Ning-Ru Zhang, Ling Xuan, Xiao-Jun Shi, Tong Zhou, Bin Chen, Jun Zhang, Hui Li

**Affiliations:** Department of Cardiovascular Disease, The First Affiliated Hospital of Bengbu Medical College, Bengbu, Anhui, China.

**Keywords:** acute coronary syndrome, gene polymorphism, Gensini score, homocysteine, methylenetetrahydrofolate reductase

## Abstract

The association between methylenetetrahydrofolate reductase (MTHFR) *C677T* polymorphism, circulating levels of homocysteine (Hcy), and the severity of coronary lesion in patients with acute coronary syndrome (ACS) remains unknown.

Consecutive ACS patients were included. MTHFR *C677T* polymorphisms were determined *via* amplification refractory mutation system-polymerase chain reaction (ARMS-PCR). Gensini scores were used to evaluate the severity of coronary lesions.

Three hundred ten ACS patients were included, and grouped according to the MTHFR *C677T* polymorphism variant: CC (n = 78, 25.2%), CT (n = 137, 44.2%), and TT (n = 95, 30.6%) groups. No significant differences were detected with respect to baseline characteristics. Patients in TT group had significantly higher Hcy, and significantly lower folic acid (FA) levels as compared with those in the other 2 groups (*P* < .05 for both). More importantly, patients with TT had more severe coronary lesions as compared with those from the other 2 groups, as evidenced by higher Gensini scores (*P* < .05 for both); however, no significant differences were observed with respect to the numbers of affected coronary arteries, or the number, length, and diameter of stents implanted in each group (*P* > .05 for all). On multivariate logistic regression analysis, presence of a T allele in MTHFR C677T was found to be independently associated with higher circulating Hcy (odds ratio [OR] = 1.06, 95% confidence interval [CI]: 1.01–1.12, *P* = .024), and higher Gensini scores (OR: 1.01, 95% CI: 1.00–1.02, *P* = .046).

MTHFR *C677T* TT polymorphism was associated with higher Hcy levels and more severe coronary lesions in patients with ACS.

## 1 Introduction

Despite significant advances in the diagnosis and treatment for atherosclerosis, coronary artery disease (CAD) continues to be one of the leading causes of morbidity and mortality worldwide.^[1,2]^ Acute coronary syndrome (ACS), characterized by rupture of vulnerable plaque in the coronary arteries and subsequent partial or complete blockage of the vascular lumen, is the most lethal manifestation of CAD.^[3,4]^ Indeed, complications of ACS, including acute cardiac failure, ventricular rupture, and malignant arrhythmia, are associated with a high mortality rate.^[5]^ The overall survival of patients with ACS has improved with the use of antiplatelet drugs and statins, and more importantly, with timely revascularization via percutaneous coronary intervention (PCI) and coronary artery bypass graft. However, primary prevention of ACS deserves more efforts as it is a fundamental strategy to lower the morbidity burden attributable to CAD.^[6]^ Therefore, early identification of patients at risk of development of severe CAD is important in clinical practice.

Evidence from experimental studies suggests that homocysteine (Hcy) may be involved in the pathogenesis of atherosclerosis.^[7]^ Moreover, epidemiological studies have proved that hyperhomocysteinemia (HHcy) is an important risk factor for stroke.^[8]^ A recent randomized controlled trial in China demonstrated that additional administration of folic acid, an intervention for lowering circulating Hcy level, to hypertensive patients on enalapril significantly reduced the risk of first stroke in patients.^[9]^ Although pilot studies have suggested that HHcy may be a risk factor for CAD,^[10]^ the association between homocysteine and the severity of coronary lesions, particularly in patients with ACS, has rarely been reported.

Accumulating evidence also suggests various gene polymorphisms may also influence the association between Hcy levels and CAD risk. Variants of *C677T* in methylenetetrahydrofolate reductase (MTHFR), an enzyme involved in the catabolism of Hcy, were shown to be associated with increased circulating Hcy, since the catabolic activity of the enzyme was reduced to 65% and 30% in CT and TT carriers as compared with that in wild type CC.^[11]^ A meta-analysis of case-control studies found that MTHFR *C677T* polymorphism was associated with risk of MI in young and middle-aged populations, particularly among Caucasians.^[12]^ However, the potential influence of MTHFR *C677T* polymorphism on the association between Hcy level and the severity of coronary lesions in patients with ACS has not been determined. Hence, we performed a pilot study to evaluate the potential association between MTHFR *C677T* polymorphism and the severity of coronary lesions in ACS patients. In addition, the potential role of circulating Hcy, folic acid (FA), and their associations with the anatomic and clinical severity of ACS were also analyzed.

## 2 Methods

### 2.1 Patients

The study protocol was approved by the Ethical Committee at the First Affiliated Hospital of Bengbu Medical College (Bengbu, China). The study included consecutive adult patients with ACS who underwent coronary angiography (CAG) percutaneous coronary intervention (PCI) at the Cardiovascular Department, the First Affiliated Hospital of Bengbu Medical College (Bengbu, China) between December 1, 2015 and April 30, 2016. Written consent was obtained from all patients prior to their enrollment. The diagnosis of ACS was in accordance to the current guidelines. All patients were of Han ethnic origin. Patients with hepatic or renal failure, severe hematopoietic diseases, severe infection, tumor or other malignant diseases, as well as diseases of endocrine system that may affect the serum Hcy, were excluded from the study. To minimize the potential influence of medications on circulating Hcy, patients receiving folic acid (FA), vitamin B6 or vitamin B12 were also excluded.

### 2.2 Baseline assessment

All patients underwent comprehensive assessments including the demographic information, medical history, and physical examination before the CAG. Medications for secondary prevention of CAD were prescribed as soon as the diagnosis of ACS was confirmed; these included aspirin, clopidogrel or ticagrelor, and statins. Other medications, including nitrates, angiotensin converting enzyme inhibitors or angiotensin II receptor blockers, and β blockers were prescribed and adjusted according to the heart rate and blood pressure of the patients. Fasting whole blood samples were collected on the second day of admission for routine blood biochemical tests, MTHFR *C677T* genotyping, and for measurement of circulating Hcy and FA levels. Hypertension was diagnosed based on the recommendation of the Chinese Guideline for the Diagnosis and Management of Hypertension (2010),^[13]^ as well as the World Health Organization and International Society of Hypertension (WHO/ISH) Guidelines for the Management of Hypertension,^[14]^ with a systolic blood pressure (BP) ≥140 mmHg and (or) a diastolic BP ≥90 mmHg. All patients were under intensive monitoring and care during their hospitalization.

### 2.3 Biochemical tests

Plasma biochemical parameters, including fasting blood glucose (FBG), total cholesterol (TC), triglycerides (TG), low-density lipoprotein cholesterol (LDL-C), high-density lipoprotein cholesterol (HDL-C), serum creatinine (SCr), serum uric acid (SUA), and C reactive protein (CRP) levels were measured using an Olympus AU5800 autoanalyzer (Olympus Co., Tokyo, Japan) at the Department of Clinical Laboratory at our institution.

### 2.4 Measurements of Hcy and FA

Circulating levels of Hcy and FA were determined via high performance liquid chromatography (HPLC) and electrochemiluminescence methods at the Cardiopulmonary Laboratory at our institution.

### 2.5 DNA extraction and determination of MTHFR C677T gene polymorphism

Deoxyribonucleic acid (DNA) was extracted from whole blood samples using a DNA extraction kit, according to the manufacturer's instructions (TIANGEN, Beijing, China). MTHFR *C677T* polymorphisms were then determined via amplification refractory mutation system-polymerase chain reaction (ARMS-PCR) at the Cardiopulmonary Laboratory at our institution. Patients were grouped according to the MTHFR *C677T* polymorphisms into CC, CT, and TT groups.

### 2.6 CAG process and the evaluation for the severity of coronary lesions

Independent cardiovascular specialists who performed the CAG and PCI procedure were blinded to the results of the MTHFR *C677T* polymorphisms tests. Results of CAG were evaluated and judged according to the criteria of ACC/AHA Guidelines for PCI (2001).^[15]^ For patients with severe coronary stenosis (>70% luminal stenosis), a drug-eluting stent (DES) was placed. Success of the PCI process was defined by a residual stenosis <20% and thrombolysis in myocardial infarction (TIMI) 3 flow. The severity of the coronary lesions was assessed using Gensini scores.^[16]^ By 2 experienced cardiovascular specialists independently and the average Gensini score was used for the subsequent analyses.

### 2.7 Statistical analyses

Continuous variables are presented as mean ± standard deviation (SD), while categorical variables are presented as frequencies and percentiles. If normally distributed, the analysis of variance (ANOVA) was applied for comparison of continuous variables among multiple groups, and Dunnett test was used to assess differences between 2 groups. However, for continuous variables associated with a non-normal distribution, Kruskal–WallisH test was used. Between-group differences with respect to categorical variables were assessed with chi-squared test. Multivariate logistic regression analysis was performed to evaluate the association between the clinical characteristics and presence of a T allele in MTHFR C677T. All statistical analyses were performed using SPSS 17.0 software (SPSS Inc., Chicago, IL). A 2-tailed *P* value <.05 was considered to be statistically significant.

## 3 Results

### 3.1 Patient characteristics and the genotype of MTHFR C677T

Overall, our study included 310 patients with ACS (179 men and 131 women) (mean age: 62.5 ± 10.8 years). Based on the results of genotyping, 78 patients were classified as CC for MTHFR *C677T* (25.2%), 137 patients were classified as CT (44.2%), and 95 patients were classified as TT (30.6%). The allele frequencies for the C and T alleles were 47.3% and 52.7%, respectively. Baseline characteristics of the included patients according to the distributions of MTHFR *C677T* gene polymorphism are shown in Table 1. No significant differences were observed with respect to the demographic characteristics (mean age, sex, and smoking habits), comorbidities (hypertension, diabetes, stroke, atrial fibrillation, and the subtypes of ACS, namely unstable angina [UA], and acute myocardial infarction [AMI]), biochemical parameters (blood glucose, blood lipids, SCr, SUA, and CRP), as well as with respect to concurrent medications (*P* > .05 for all).

**Table 1:**
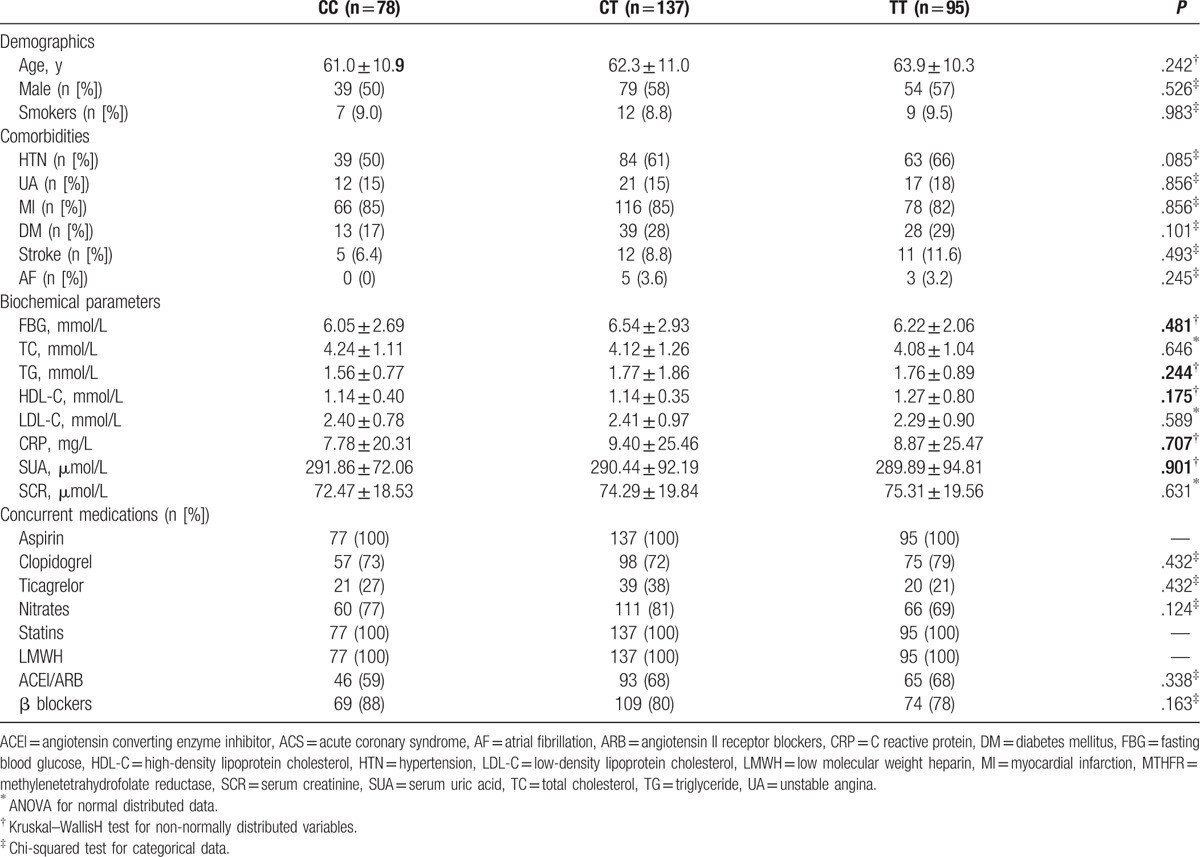
Baseline characteristics of the included ACS patients according to MTHFR *C677T* polymorphism.

### 3.2 Circulating Hcy and FA according to MTHFR C677T polymorphism

**Figure 1:**
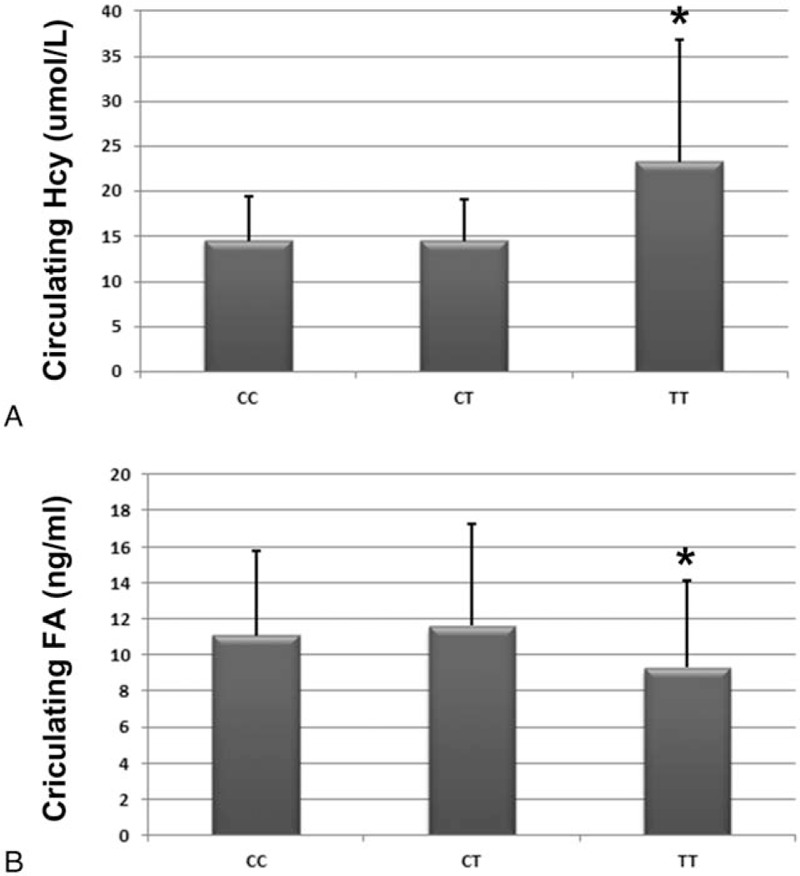
Circulating Hcy and FA levels according to the distribution of MTHFR *C677T* gene polymorphism in patients with ACS. (A) Circulating levels of Hcy as grouped by MTHFR *C677T* gene polymorphism; (B) circulating level of FA as grouped by MTHFR *C677T* gene polymorphism; ^∗^*P* < .05 as compared with the CC or CT group. ACS = acute coronary syndrome, Hcy = homocysteine, FA = folic acid, MTHFR = methylenetetrahydrofolate reductase.

Patients from different groups according to the MTHFR *C677T* polymorphism had different baseline levels of circulating Hcy (*P *<* *.001 on ANOVA) and FA (*P* = .004 on ANOVA). Specifically, patients in the TT group showed significantly higher circulating Hcy levels (23.25 ± 13.58 μmol/L) as compared with those in patients in the CC (14.52 ± 4.86 μmol/L) and CT (14.53 ± 4.55 μmol/L) groups (*P* < .05; Fig. 1A). The converse was true with respect to FA levels; FA levels in the TT group (9.28 ± 4.83 ng/mL) were significantly lower than those in the CC (11.08 ± 4.71 ng/mL) and CT (11.59 ± 5.68 ng/mL) groups (*P* < .05; Fig. 1B).

### 3.3 Gensini scores according to MTHFR C677T polymorphism

**Figure 2:**
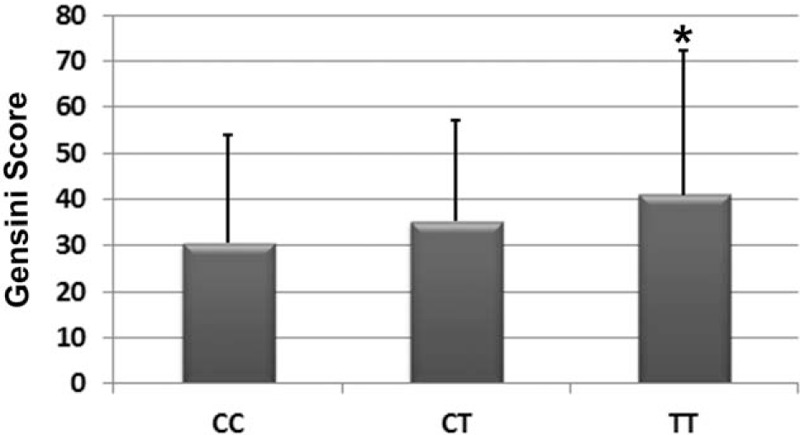
Distribution of MTHFR *C677T* gene polymorphism and the severity of coronary lesions in ACS patients as evaluated by the Gensini scores. ACS = acute coronary syndrome, MTHFR = methylenetetrahydrofolate reductase.

Gensini scores in the TT group (40.92 ± 31.41) were significantly higher than those in the CC (30.60 ± 23.38) and CT (35.18 ± 25.90) groups (*P* < .05; Fig. 2). These results suggest that ACS patients with TT genotype of MTHFR *C677T* had more severe coronary lesions as compared with their counterparts with the other 2 genotypes.

### 3.4 Perioperative characteristics according to MTHFR C677T polymorphism

PCI perioperative characteristics of ACS patients with different MTHFR *C677T* polymorphisms are shown in Table 2. No significant differences were observed with respect to the proportions of patients that received stent implantation, the number of coronary arteries affected, the number of stents implanted, or with respect to the diameters and lengths of the stents used (*P* > .05 for all).

**Table 2:**
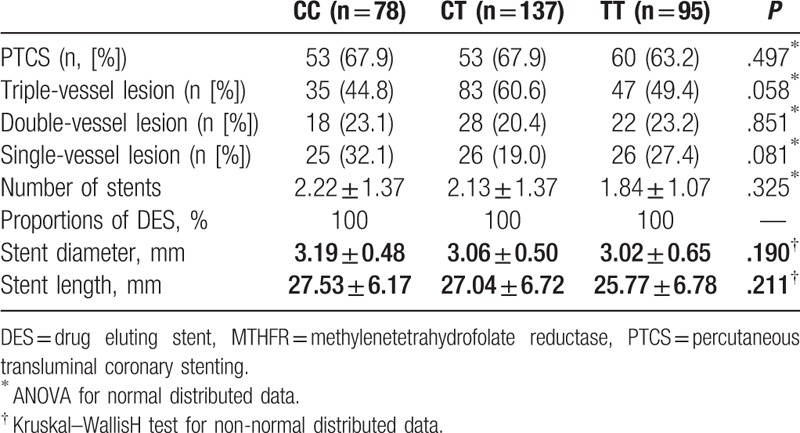
Perioperative characteristics according to MTHFR *C677T* polymorphism.

### 3.5 Prevalence of MTHFR C677T polymorphism according the subtype of ACS

Distribution of MTHFR *C677T* genotypes and other related risk factors according the subtype of ACS are shown in Table 3. Although no significant differences with respect to distribution of MRHFR *C677T* genotypes were observed between patients with UA and those with AMI, circulating Hcy levels as well as the levels of FBG, TG, and CRP were significantly higher in patients with AMI (*P* < .05 for all). These results suggest that patients with AMI may have more remarkably increased Hcy, and more severe metabolic imbalance, and enhanced inflammatory response.

**Table 3:**
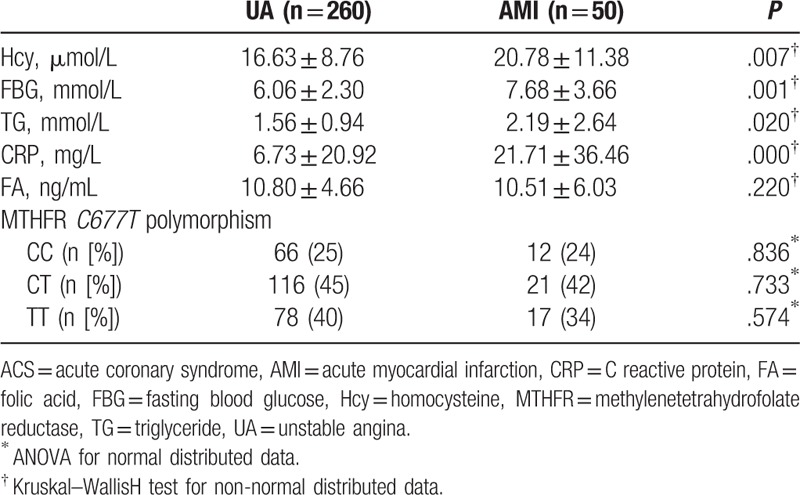
Clinical characteristics and MTHFR *C677T* polymorphism according to the subtype of ACS.

### 3.6 Patient characteristics and the presence of a T allele in MTHFR C677T

We performed multivariate logistic analysis to explore the potential association between patient characteristics and the presence of a T allele in MTHFR C677T (Table 4). The results showed that presence of a T allele in MTHFR C677T was independently associated with higher circulating Hcy (odds ratio [OR] = 1.06, 95% confidence interval [CI]: 1.01–1.12, *P* = .024), and higher Gensini scores (OR: 1.01, 95% CI: 1.00–1.02, *P* = .046).

**Table 4:**
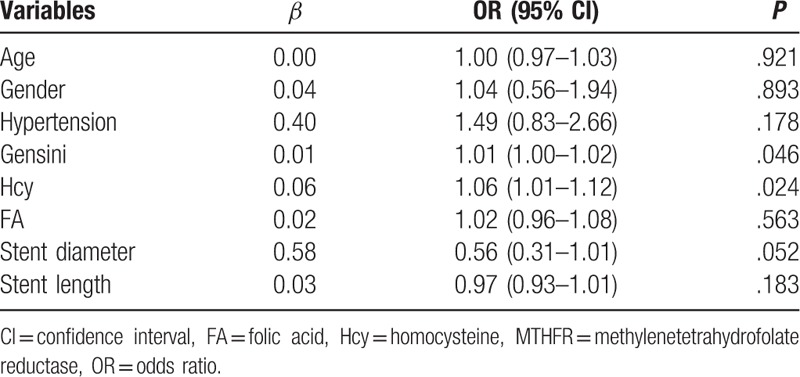
Associations between patient characteristics and the presence of T allele of the MTHFR *C677T* gene polymorphism: results of the multivariable logistic regression.

## 4 Discussion

In a cohort of ACS patients who had undergone CAG, we found that patients with TT variants of the MTHFR *C677T* polymorphism were associated with significantly higher circulating Hcy and significantly lower FA levels as compared with those with CC or CT variants. In addition, patients with TT variants had significantly higher Gensini scores as compared with that in patients with CC or CT, which indicates that the TT variants of the MTHFR *C677T* polymorphism was associated with more severe coronary lesions, although the baseline characteristics and other perioperative characteristics were comparable between the groups. Moreover, presence of a T allele in MTHFR C677T was independently associated with higher circulating Hcy and higher Gensini scores. Collectively, these results suggest that the TT variants of the MTHFR *C677T* polymorphism may be an important marker of CAD owing to its’ association with increased circulating Hcy and more severe coronary lesions.

Results of our study indicate that the TT variant of the MTHFR *C677T* polymorphism was associated with more severe coronary lesions in patients with ACS. These results are consistent with those of some earlier studies. In a previous case-control study, a T genotype of MTHFR *C677T* gene polymorphism was associated with higher incidence rate of vulnerable plaque and higher Hcy level, which were both considered as risk factors for more severe coronary artery disease.^[17]^ In another case-control study (208 controls and 200 healthy subjects), the frequency of T genotype of MTHFR *C677T* gene polymorphism among patients with CAD was higher than that in controls.^[18]^ More importantly, the T genotype was more prevalent in ACS patients as compared with that in patients with stable CHD.^[18]^ Also, higher Hcy was noticed in CHD than controls. Our study further extended these results by using the overall severity of the coronary lesions as evaluated by Gensini score of CAG results. Overall, these results indicate that the T genotype of MTHFR *C677T* gene may be a risk factor for the prevalence of more severe coronary lesions, which is probably related to a higher circulating Hcy value in these patients.

An earlier small-scale study of 53 patients with CAD showed that higher circulating Hcy was related to the severity of coronary disease, as evaluated by atheroma burden index.^[19]^ These results were further confirmed in subsequent case-control studies which suggest a possible independent association between Hcy and severity of CAD.^[20,21]^ A recently published study from China that included 292 CAD patients of different categories, and 100 non-CAD controls concluded that HHcy is independently associated with the severity of CHD, and significantly correlated with low folic acid levels in CAD patients.^[10]^ The above findings suggest a possible association between T genotype of MTHFR *C677T* gene, higher circulating Hcy, lower FA levels, and more severe coronary lesions. These results warrant interventions aimed at lowering of Hcy levels in high-risk patients, particularly in those with T genotype of MTHFR *C677T* gene. Although previous randomized controlled trials (RCTs) of FA supplementation aimed at lowering Hcy levels did not show significant improvement in cardiovascular outcomes,^[22,23]^ these studies were limited to low-risk patients; moreover, studies that have evaluated the genotype of MTHFR *C677T* gene have been rare. In fact, higher Hcy levels in patients with high-risk CAD have been shown to be associated with poor outcomes. In a cohort of 587 patients with CAG-confirmed CAD (318 underwent CABG and 120 received PCI), higher baseline Hcy was shown to be a strong predictor of mortality over a follow-up duration of 4.6 years.^[24]^ Therefore, we hypothesize that interventions aimed at lowering Hcy levels in high-risk patients (such as ACS patients after PCI with T genotype of MTHFR *C677T* gene) may improve cardiovascular outcomes. Further studies are needed to evaluate the above hypothesis.

Although the potential mechanisms underlying the association between higher circulating Hcy and more severe coronary lesions are not fully understood, results from experimental studies may provide some clues. Early in vitro studies suggest that Hcy may cause endothelial insult via enhanced inflammatory and oxidative stress related response.^[25]^ Subsequent studies found Hcy may induce apoptosis of endothelial progenitor cells via enhancement of endoplasmic reticulum stress-mediated activation of caspase-3.^[26]^ Moreover, HHcy was also shown to be related to impaired endothelial function as evidenced by reduced levels of nitric oxide,^[27,28]^ which is a key factor involved in the pathogenesis and progression of atherosclerotic vascular disease. Besides, Hcy may also deteriorate the status of CAD via its interaction with cardiomyocytes. In a rat model of myocardial ischemia, Hcy was shown to reduce homing of cardiac stem cells, and subsequently restrain myocardial repair mediated by these cells.^[29]^ Moreover, HHcy was shown to lead to dysfunction and apoptosis of cardiomyocytes, an effect that was mediated via other signaling pathways, such as activation p38 MAPK.^[30,31]^ Therefore, current evidence suggests that HHcy may aggravate CAD via its interaction with both the endothelial cells and cardiomyocytes.

Some limitations of our study need to be considered while interpreting the results. Firstly, since the sample size of the included patients was relatively small, we did not perform a multivariate analysis of the association between the distribution of TT variants of the MTHFR *C677T* polymorphism and the severity of the coronary lesions. Therefore, we were not able to conclude that the TT variant of the MTHFR *C677T* polymorphism is an independent determinant of the severity of coronary lesions in patients with ACS. Further studies with adequate sample size are needed to determine the influence of conventional risk factors for CAD on the observed association of TT variants with severity of CAD. Further, whether the association between the TT variant and severity of coronary lesion persists after adjusting for circulating Hcy levels also needs to be determined. Secondly, similar to other observational studies, our study design does not allow us to draw causal inferences from the observed association between MTHFR C677T polymorphism, higher circulating Hcy, and more severe coronary lesions. Moreover, we focused our study on the outcome of severity of coronary artery disease. Whether MTHFR *C677T* polymorphism is associated with other clinical outcomes, such as the risk for the development CAD, and prognosis of CAD patients also deserves evaluation. Finally, although our study confirmed that patients with higher circulating Hcy are associated with severer coronary lesions, the efficacy of Hcy-lowering therapy for the prevention and treatment of CAD remains to be evaluated. Current evidence from meta-analyses of RCTs does not support an effective role of Hcy-lowering therapy for reducing the risk of cardiovascular events.^[22,23]^ However, whether Hcy-lowering interventions may improve the cardiovascular prognosis in high-risk population, such as those with ACS after PCI, should be further investigated.

In conclusion, MTHFR *C677T* TT polymorphism was associated with higher Hcy levels and more severe coronary lesions in patients with ACS. Whether Hcy-lowering therapies could improve clinical outcomes in these patients requires further investigation.
